# Can ill-structured problems reveal beliefs about medical knowledge and knowing? A focus-group approach

**DOI:** 10.1186/1472-6920-9-62

**Published:** 2009-09-23

**Authors:** Ann Roex, Geraldine Clarebout, Valerie Dory, Jan Degryse

**Affiliations:** 1 Department of General Practice, KULeuven, Kapucijnenvoer 33, Blok J, Bus 7001, 3000 Leuven, Belgium; 2 Center for Instructional Psychology and Technology, KULeuven, Vesaliusstraat 2, 3000 Leuven, Belgium; 3 Département de Médecine Générale, Université catholique de Louvain, Avenue Mounier 53 (bte 5360), 1200 Brussels, Belgium; 4 Department of General Practice, KULeuven Kapucijnenvoer 33, Blok J, Bus 7001, 3000 Leuven, Belgium

## Abstract

**Background:**

Epistemological beliefs (EB) are an individual's cognitions about knowledge and knowing. In several non-medical domains, EB have been found to contribute to the way individuals reason when faced with ill-structured problems (i.e. problems with no clear-cut, right or wrong solutions). Such problems are very common in medical practice. Determining whether EB are also influential in reasoning processes with regard to medical issues to which there is no straightforward answer, could have implications for medical education. This study focused on 2 research questions: 1. Can ill-structured problems be used to elicit general practice trainees' and trainers' EB? and 2. What are the views of general practice trainees and trainers about knowledge and how do they justify knowing?

**Methods:**

2 focus groups of trainees (n = 18) were convened on 3 occasions during their 1^st ^year of postgraduate GP training. 2 groups of GP trainers (n = 11) met on one occasion. Based on the methodology of the Reflective Judgement Interview (RJI), participants were asked to comment on 11 ill-structured problems. The sessions were audio taped and transcribed and an adapted version of the RJI scoring rules was used to assess the trainees' reasoning about ill-structured problems.

**Results:**

Participants made a number of statements illustrating their EB and their importance in clinical reasoning. The level of EB varied widely form one meeting to another and depending on the problem addressed. Overall, the EB expressed by trainees did not differ from those of trainers except on a particular ill-structured problem regarding shoulder pain.

**Conclusion:**

The use of focus groups has entailed some difficulties in the interpretation of the results, but a number of preliminary conclusions can be drawn. Ill-structured medical problems can be used to elicit EB. Most trainees and trainers displayed pre-reflective and quasi-reflective EB. The way trainees and doctors view and justify knowledge are likely to be involved in medical reasoning processes.

## Background

Questions such as 'How certain is knowledge?', 'Is knowledge definitely right or wrong?', 'How do I justify what I know about something?' play an important role in the way one process and reflect upon (new) information[1] Suppose Doctor A is convinced that all phenomena can be explained by a simple cause-effect relationship. She thinks, for example, that breast cancer is caused by environmental factors, a belief she holds as a result of reading a newspaper article (justification by authorities) and is little inclined to investigate further. Doctor B has read the same article, but suspects that other factors such as genetic predisposition, smoking, hormonal contraceptives play an important role. For Doctor B, there is no clear-cut limit between right and wrong information. She is interested in the reason for this discrepancy in etiological explanations and undertakes a search of medical websites. What information is she going to consider and why? How does she relate her existing knowledge to the information found on the web? How is she going to justify her final opinion on this matter? .... (example based on [2])

The epistemological beliefs (EB) research offers a framework for studying these questions. EB has proved a popular research topic in educational psychology due to the underlying assumption that understanding these beliefs and their influence may help educators to improve teaching methods[3]

EB, have been defined as the cognitions (i.e., ideas) that individuals hold about knowledge and knowing [4]. Within the existing body of research, 2 major categories of EB recur: the view on knowledge (is knowledge simple or complex?, is knowledge certain or not?) and the justification of knowing (how do we justify what we know?) [5-9]

Within domains other than medicine, EB have been linked to strategy use, for instance, information seeking strategies [1], text comprehension and text processing [10], reasoning and problem solving [11], academic performance [12,13] and motivation [11,14].

There are several reasons to suspect that EB may play an equally important role in medical practice and education. Firstly, there is general agreement that EB are associated with learning[9,12,15] Self-assessment and life-long-learning have been given a central place in current definitions of 'medical competence'[16,17] Medical doctors are expected to continuously question their own knowledge, and to search for and critically appraise new information. EB may play an important role in this process. Secondly, Knight and Mattick have found that medical students' EB are involved in their professional identity formation[18] Thirdly, a number of authors have emphasized the uncertain nature and context-dependency of medical knowledge. Sturmberg and Martin argue that the nature of medical knowledge is inherently uncertain and thus needs to be approached in a flexible, context-dependent way[19] A straightforward, universal application of existing evidence in practice is often difficult[20,21] EB research offers a framework for studying how individuals view these fundamental aspects of knowledge and knowing. Fourthly, individuals' EB are said to come to the surface when confronted with ill-structured problems (questions to which no absolutely right or wrong answers is available). As physicians, we are often confronted with such questions in medical practice, and several authors have argued the importance of including these in medical curricula and assessment[22,23]

Understanding the relevance of EB and their actual role in learning, identity formation and clinical reasoning may help medical educators to design instructional environments which enable students to acquire these skills more efficiently.

### The 'Reflective Judgement' model

King and Kitchener initiated one major line of research into EB[11] They proposed a model of 'Reflective Judgement' which describes the development of individuals' EB in 3 broad phases or 7 stages (table 1). King and Kitchener use two dimensions to describe EB: the way students view knowledge and the way students justify knowing.

**Table 1:** Summary of King and Kitchener's Seven Stages of Reflective Judgement[11]

**Stage**	**View of knowledge**	**Concept of Justification**	**Consequences**	**Level**
1	"What I have seen is true" Knowledge is absolute and predetermined.	Beliefs need no justification.	Failure to understand that 2 people can disagree about something	
2	Knowledge is available through the senses or via authority figures. Knowledge is absolutely certain or certain but not immediately available.	Beliefs are unjustified or justified by beliefs of authorities	Dogmatic and naïve views - belief in right and wrong answers	**Pre-reflective thinking**
3	Knowledge is absolutely certain or temporarily uncertain (future data will demonstrate the truth).	Predominance of personal opinions in areas of temporary uncertainty. Reference to authorities' views in areas of certainty.	Confusion when asked to make decisions about problems for which no absolute answers exist.	

4	Knowledge is understood as an abstraction. Knowledge in certain fields will never be certain. Knowledge claims are idiosyncratic to the individual.	Arguments and choice of evidence for justification of beliefs are idiosyncratic.	New tolerance of alternative perspectives.	**Quasi-reflective thinking**
5	Knowledge is contextual and subjective because it is filtered through a person's perceptions and criteria for judgement.	Justification by rules of inquiry for that context.	Different views are seen as potentially legitimate interpretations of issues. What is missing is the ability to compare and contrast evidence across contexts.	

6	Inadequacy of purely contextual and subjective knowing becomes apparent. No understanding of larger system of knowing in which comparisons are embedded.	Justification by comparing evidence and opinions and construction of solutions (based on weight of evidence, utility of the solution,...)	Conclusions remain limited and situational.	**Reflective** **thinking**
7	Belief that while reality is never a given, interpretations of evidence and opinion can be synthesized into epistemically justifiable conjectures about the nature of the problem. (recognition of its temporary character)	Beliefs are justified probabilistically on the basis of a variety of interpretive considerations.	Possession of a general framework about knowledge and justification. Conclusions are defended as representing the most complete, plausible or compelling understanding of an issue on the basis of the available evidence.	

During the first phase ('pre-reflective thinking'), individuals typically view knowledge as absolute and observable. As a result of this conception of knowledge they do not see the need to justify their beliefs, and they fail to understand that experts disagree on a particular issue. In the last phase, called reflective thinking, individuals, when confronted with an ill-structured problem, go through a process of inquiry during which they first seek possible solutions and consequently evaluate these according to standards from existing evidence and the characteristics of the context in which the problems were presented. The evaluation is typically dynamic in the 7th stage of reflective judgment. Knowledge is re-evaluated when new evidence is available and when the context changes.

King and Kitchener developed the 'Reflective Judgement Interview' (RJI) to map out beliefs about the nature of knowledge and the arguments for justification of knowing. During such a one-to-one interviews, the questioner presents the subject with a number of ill-structured problems and focuses the subject's reflection on the key aspects of EB (using what they refer to as 'standard probe questions': table 2). Trained raters later assess the transcripts using a set of detailed scoring rules. Using the RJI, King and Kitchener collected many data on 1700 subjects (of varying ages from early adolescence through to adulthood). This resulted in interesting findings on developmental patterns, and on the link between reflective judgement on the one hand and intellectual and character development on the other[11]

**Table 2:** Standard Probe questions[11]

What do you think about these statements?	How did you come to hold that point of view?
On what do you base that point of view?	Can you ever know for sure that your position on this issue is correct? How and why not?
When 2 people differ about matters such as this, is it the case that one opinion is right and one is wrong?	If yes, what do you mean by 'right'? If no, can you say that one opinion is in some way better than the other? What do you mean by 'better'?
How is it possible that experts in the field disagree about this subject?	How is it possible that people have such different points of view about this subject?

There are a number of reasons why the use of the RJI is particularly appealing in an initial exploration of medical trainees' EB. There has been growing attention paid in medical education to the ill-structured nature of medical knowledge, encouraging medical educators to search for appropriate tools to teach students how to deal with uncertainty, complexity, context-dependency etc. To do so however requires further understanding of factors involved in reasoning on ill-structured problems. The RJI model offers a way of looking at reasoning processes related to such problems and might lead to a clearer picture on the role of EB. Secondly, the ill-structured problems which King and Kitchener present during their semi-structured interviews can easily be replaced by 'clinical vignettes', that is a patient description with important clinical information and an underlying problem (key-features[24]). Thirdly, the use of clinical vignettes and standard probe questions (table 2) is relatively straightforward and, if proven to lead to the expression of medical students' EB, could easily be used for educational interventions.

The aims of this preliminary study were to determine whether the presentation of ill-structured problems could lead to the expression of medical trainees' EB, and to subsequently study these beliefs qualitatively.

#### Research questions

1. Can ill-structured problems be used to elicit general practice trainees' and trainers' EB?

2. If so, what are these EB: how do trainees and trainers view knowledge and how do they justify knowing?

## Methods

### Participants

General practice training in Flanders (the Dutch-speaking part of Belgium) takes place at post-graduate level and lasts 3 years. Two existing study groups of trainees in their 1^st ^year of GP training were invited to participate in the study (n = 11 and n = 7). The choice of these particular 2 groups of students was based on organisational considerations. Each group took part in 3 focus group sessions at the beginning, in the middle and at the end of this 1^st ^year. Participation was on a voluntary basis. The trainees were told that the meetings formed part of a process of evaluation of the curriculum and that their contribution to it would not be linked to other assessments. They were offered a sandwich lunch at every meeting, and 2 film tickets (by way of incentive) once they had attended the 3 meetings. Two focus group sessions were held with experienced GP trainers (n = 11).

### Format

During each meeting the participants were presented with one of King and Kitchener's ill-structured problems and 3 (newly developed) medical ill-structured problems (table 3). The medical problems (vignettes) were developed by the first author and validated by the second author. We repeatedly emphasized that we were interested in the reasoning processes behind the students' scientific arguments and focused their answers on the standard probe questions (table 2).

**Table 3:** Ill-structured problems:

**Example of a** **non-medical problem** (from King and Kitchener)[11]	"Most historians claim that the pyramids were built as tombs for kings by the ancient Egyptians, using human labor, and aided by ropes, pulleys, and rollers. Others have suggested that the Egyptians could not have built such huge structures by themselves, for they had neither the mathematical knowledge, the necessary tools, nor an adequate source of power."
**Example of a** **medical problem:** (NHG standaard, Schouderklachten)	"When a patient presents with shoulder pain, one must check the active and passive abduction and exorotation of both arms. Other tests, such as resistance tests, horizontal adduction and passive endorotation are unnecessary, because they do not provide relevant information for management."

11 different (7 medical & 4 non-medical) ill-structured problems were used. All of these were presented to the students and 8 of these were presented to the trainers.

### Coding & data-analysis

All sessions were audio taped and transcribed. A framework for coding (based on the coding system from the RJI) was developed by the researchers, then discussed and validated by an expert group of medical educators. Coding was done independently by 2 researchers. All statements on which the coders disagreed were discussed until consensus on labelling was reached. Results were analysed using Atlas-ti software[25]. Fifteen hours of tape were analyzed in total.

### Ethical considerations

The research project was carried out with the approval of the Educational Board of the Department of General Practice of the K.U.L. ('Permanente Onderwijscommissie') according to the current procedure at our University. No further ethical approval was sought in view of the formative nature of participation. Participation was on a voluntary basis. The interviewer and coders were not involved in certification examinations. Participants were informed of the purpose of the study. The decision to take part was taken as proof of consent.

## Results

All but one of the trainees invited (n = 18/19) and all of the GP trainers invited (n = 11/11) agreed to participate in the focus group meetings. One trainee did not attend the second meeting, but the others were present at all 3 meetings.

### Can ill-structured problems be used to elicit general practice trainees' and trainers' EB?

**Figure 1:**
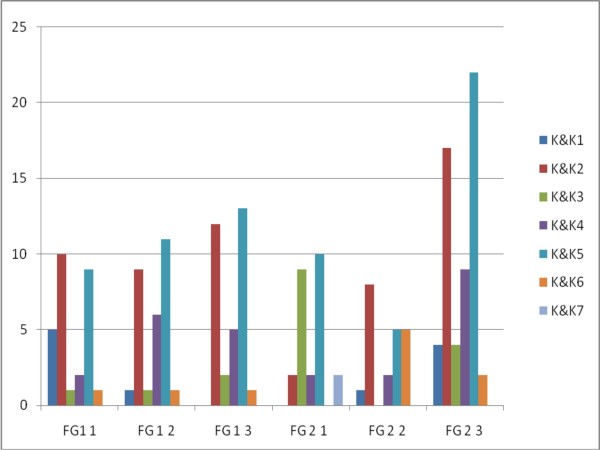
**Number of EB's expressed per focus group meeting with the trainees**. FG 1 1: refers to the first meeting of the focusgroup 1. FG 2 1: refers to the first meeting of the focusgroup 2. K&K1-7: refers to the expressed level of EB as defined in the King and Kitchener's Reflective Judgement Interview [11].

**Figure 2:**
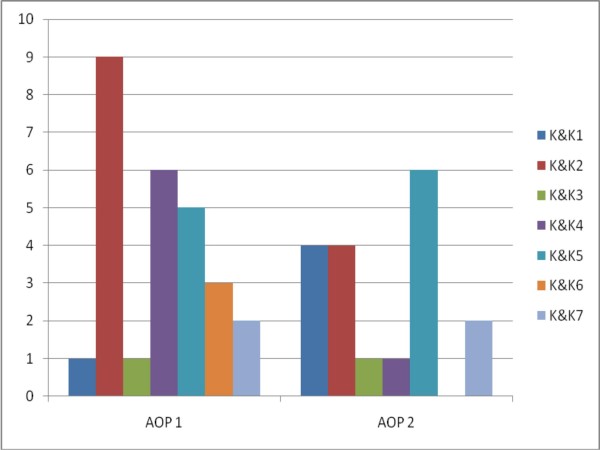
**Number of EB's expressed per focus group meeting with the medical trainers**. AOP 1: refers to the meeting with the first group of GP trainers. AOP 2: refers to the meeting with the second group of GP trainers. K&K1-7: refers to the expressed level of EB as defined in the King and Kitchener's Reflective Judgement Interview [11].

We used King and Kitchener's framework to analyze the data, and found that it did allow us to code a considerable number of statements made by participants. Figures 1 and 2 illustrate the number of statements expressing EB and demonstrate that both trainees and trainers did express a wide range of EB at different levels. The coding and recoding of these resulted in an interrater reliability of 0.851 (Spearman's rank order correlation coefficient with p < 0.01), an interrater agreement of 81.4% and a Weighted Kappa of 0.740.

### What were participants' views on knowledge?

Participants expressed EB reflecting different stages of development.

One of the vignettes argues that depression should be considered as a disease in view of its association with an imbalance in the levels of neurotransmitters in the brain.

One of the trainers answered:

"That is absolutely right. When we think about the serotonin involvement, our daily experience has taught us that we get very good results when we give serotonin reuptake inhibitors. We all agree on that."

When asked why there are different opinions on the same issue, one trainee answered that:

'I think that there is still a lot that is unknown in that field....'

According to King & Kitchener's staging of EB, these two statements indicate EB at the first stage of pre-reflective thinking: the belief that knowledge is certain and available through direct observation. In this case, the trainee assumed knowledge to exist absolutely, and that good and thorough observation would lead to the correct information. Neither of them seemed to consider alternative points of view at this stage.

Another trainee saw the different points of view as more context dependent and subjective (as filtered through a person's perceptions). This is what King and Kitchener describe as the quasi-reflective phase (see table 1).

"It just depends on how you look at it. When we, for example, look at the domain of psychology from the medical perspective, we look at it differently than people who look at it from another field of interest."

This trainee had not quite reached the reflective phase of EB, because he did not seem able to select a single point of view as more plausible. Rather, he was convinced that different opinions result from different but equally legitimate interpretations of the same observation.

Another trainee translated these beliefs into the idea that knowledge is uncertain and that it is impossible to know for certain. He found this thought deeply disturbing:

*'You don't know, and we will probably never really know. It (the construction of Egyptian pyramids) happened too long ago. But if that's what we have to tell the patients*...

The shoulder vignette states that in order to select an appropriate management plan for patients with acute shoulder pain, only a minimal number of clinical tests are required. In discussing this vignette, trainers pondered the context-dependency of knowledge (evidence), its evolutive nature and the need to select the most plausible interpretation of knowledge in a given situation. These are arguments suggesting reflective levels of EB. For example:

"That position is taken by the Dutch Guidelines because their consultations last 10 minutes per patient... Those are indeed the most important tests given that timeframe."

Or:

"How do I explain that experts differ on this matter? Firstly, the way you decide to write guidelines. Secondly, the strategy the Dutch GP uses to make the guidelines. They only keep data of which they are sure there is evidence... Maybe we shouldn't forget that the guidelines are 6 years old. I don't know whether the guidelines would be the same if they were rewritten today."

### How did the participants justify knowing?

In the pre-reflective phase, knowledge requires no justification (stage 1) or is justified through the opinion of experts or authorities (stages 2 and 3). In our study, trainees and trainers very often justified knowledge through the views of authorities:

'I think I would first prescribe an ACE-inhibitor, because that's what I learnt during the endocrinology course and during my training.'

'Even if it no longer recommended, my training experience means that I still think it's important'

*'From the courses, particularly in the 4*^*th *^*year. From cases and pharmacology etc.'*

The subjects' own experiences were also often dominant in the justification of choices they made.

"I have worked on the urology ward, and that is where I have seen that..."

"In general practice, decisions are more based on one's own experience"

"During my practical training, I saw that the GP prescribed antibiotics straight away, because he knows the situation from his experience. So at first I will stick to the guidelines, but later on my experience will play a more important role."

Justification in the reflective stages is a result of a variety of considerations: weight of evidence, explanatory value of interpretations etc.

A GP trainer referred to the weight of evidence which is different for diagnostic research and for RCTs (Randomized Clinical Trials):

*"I am sometimes irritated, especially with these studies (the guideline on acute shoulder pain), because absence of evidence does not necessarily mean that these tests are clinically irrelevant. The major problem with diagnostic tests is that it is very difficult to standardize the clinical examination. It is much easier to standardize RCTs. But if you decide to be guided by existing evidence, one indeed has to agree with this statement"*.

Similarly, in one of the groups, the conversation was repeatedly focused on the difficulty of applying statistical group results to individual patients:

*"If you look at studies which start with 1000 subjects, the ones who react to the placebo and the ones with co-morbidity are excluded. ... And how well do, let's say, 100 'ideal' patients that *are *included represent the rest of the population? They are used in the guidelines that we have to interpret, but three quarters of the population doesn't fit in this model !*

## Discussion

### Main findings

The study demonstrates that medical trainees and practitioners, when confronted with ill-structured problems, can express a range of EB. In this study, both GP trainees and trainers displayed EB that were for the most part pre- and quasi-reflective.

Niessen has outlined the grounding choices that have to be made within frameworks for studying EB[26] One of the questions raised is to ask how explicit or implicit EB are assumed to be (or to what extent people are able and prepared to talk about their EB). The choice of the RJI as a method in this project was based on the hypothesis that EB on medical knowledge can be made explicit in the context of solving ill-structured problems.

Trainees in the second focus group expressed a higher number of EB during the last meeting (figure 1). These EB did not differ from those expressed earlier in terms of stages of development. Several explanations may account for this finding. Participants may have been more familiar with the type of problems presented in the last meeting, social interactions may have been different during that meeting, or indeed participants may have become more conscious of their own EB or more articulate at expressing them.

Both trainees and trainers were well informed about the guideline on 'Acute Shoulder Pain', but trainers were more critical towards its content. On this problem, trainers systematically reached levels of more relativistic EB than trainees. The recommendations on the clinical examination of the shoulder have, due to changes in the available evidence and in contextual factors, altered drastically in the most recent version of this guideline. As a result, trainers may therefore not only have acquired more knowledge on this particular topic, but may also have learned to articulate and nuance the different points of view.

The trainers did not seem able to transfer their more developed EB on the shoulder problem to other medical problems. In line with findings in medical reasoning (content-specificity[27]), EB may be dependent on prior knowledge, in other words, the expression of (more relativistic) EB on a particular topic may require individuals to possess sufficient topic-related knowledge. King and Kitchener assumed the generic nature of reflective thinking (i.e. the way students believe in the nature of knowledge is similar across different domains), and their research brought forward arguments in favour of this domain independency. [11] Many other researchers have been able to demonstrate that EB can differ according to the domain [28-30] The results of this study do not allow us to determine whether medical students hold domain-general EB or whether they vary from one problem to another. Further research will have to answer questions such as 'Can medicine be considered as a single domain?' and 'Are EB case-specific?'.

It is likely that the possession of knowledge, as well as the awareness of one's own EB, the ability to express one's EB in an articulate fashion, and the context in which problems are presented, determine which EB come to the surface. This lends support to Hammer, Elby and Louca's theory on EB (so called 'cognitive resources') which stipulates that every individual possesses a whole range of 'cognitive resources' (i.e., a range of EB). [31,32]. Depending on the nature of the stimulus and the context in which it is presented, different resources are believed to be activated resulting in different behaviour (i.e. different way of solving ill-structured problems). Most medical educators will recall situations in which trainees function at the highest levels of reflection (e.g. during a lesson on evidence-based medicine), and other situations in which the same trainees only seem able to use dualistic frameworks (e.g. when faced with a patient). More research needs to be done to unravel the particular nature of individuals' EB in medical settings.

### Strengths and limitations

In line with the development of the new, domain-specific frameworks for EB, the need has arisen to include different methods of assessment in the study of EB [4,31,32]. This focus group approach observed trainees' reasoning processes within the format of a meeting, and with the use of ill-structured problems as stimuli. The EB literature has repeatedly emphasized that there are differences between the EB expressed through the subjects' behaviours (enacted EB) and the beliefs they claim to have (professed EB)[33] Although the statements were carefully selected in order to make the context and problem as realistic as possible (real patient problems), it remains unclear to what extent the expressed beliefs are the participants' real beliefs. Obviously, the observation of these same trainees, within more natural settings (during their actual practice) and confronted with 'natural' stimuli (real patients) could reveal useful additional information.

The possible role of social desirability in the answers was tempered by choosing a member of staff who is not involved in any teaching or assessment procedures to moderate focus groups. Furthermore, there was a considerable variation in level of EB expressed per problem. This suggests that students' answers were not influenced by their peers' opinions. However replicating this study using one-to-one in-depth interviews might prove useful in this respect.

The groups were not stratified samples of the study population, but study groups. This choice, was mainly a practical one, and is recognized as a valid way of sampling for illuminating cognitive processes. [34]

The statements expressed by the participants suggest that EB are indeed engaged in clinical reasoning. The interpretation of the results of this study regarding the actual nature and levels of the beliefs expressed and regarding their role in clinical settings remains unclear. Nevertheless, we do think that this study provides groundwork upon which more detailed studies can be developed. The challenges in future research will be to take into account a number of pitfalls inherent both to the study of medical educational research and to the field of EB [35], but also to focus on the core question as to how EB are related to medical performance. Do physicians with more reflective EB solve medical problems more accurately? Do more reflective EB make physicians perform better? Could educational interventions targeting EB lead to better doctors?

This study illustrates how the presentation of ill-structured medical problems, guided by a number of probe questions, leads to the expression of EB and permits their characterization. If EB are indeed confirmed to be influential in the solution of medical problems, this approach could prove to be a very valuable starting point for improving clinical reasoning.

## Conclusion

This study has demonstrated that medical ill-structured problems can elicit EB. Medical trainees' and trainers' EB were similar and varied considerably from one problem to another.

The focus group approach has raised the question of whether EB have a generic nature, or whether context, knowledge, awareness and the ability to formulate one's EB in an articulate manner might also play a role in the expression of EB. These findings indicate the merits of the development of an appropriate set of measures of EB, the design of additional quantitative studies and intervention studies.

## Abbreviations

(EB): Epistemological Beliefs; (RJI): Reflective Judgement Interview; (GP): General Practitioner.

## Competing interests

The authors declare that they have no competing interests.

## Authors' contributions

AR and JD participated in the design and the execution of the study. All authors participated in the interpretation of the study results. AR drafted the manuscript. JDe, VD and GC contributed to the improvement of the manuscript. All authors read and approved the final manuscript.

## Acknowledgements

No acknowledgments.

## Pre-publication history

The pre-publication history for this paper can be accessed here:


